# Activating somatic and germline *TERT* promoter variants in myeloid malignancies

**DOI:** 10.1038/s41375-020-0837-6

**Published:** 2020-05-04

**Authors:** Valeria Nofrini, Caterina Matteucci, Fabrizia Pellanera, Paolo Gorello, Danika Di Giacomo, Anair Graciela Lema Fernandez, Carlotta Nardelli, Tamara Iannotti, Lucia Brandimarte, Silvia Arniani, Martina Moretti, Alessio Gili, Giovanni Roti, Valeria Di Battista, Simona Colla, Cristina Mecucci

**Affiliations:** 1 University of Perugia, Section of Hematology and Center for Hemato-Oncology Research (C.R.E.O.), Perugia, Italy; 2 Public Health Section, Department of Experimental Medicine, University of Perugia, Perugia, Italy; 3 Hematology and Bone Marrow Transplantation Unit, University of Parma, Parma, Italy; 4 Department of Leukemia, The University of Texas MD Anderson Cancer Center, Houston, TX USA

**Keywords:** Diseases, Myelodysplastic syndrome, Diseases, Myelodysplastic syndrome

## To the Editor:

*TERT* gene is encoding for the telomerase enzyme catalytic subunit, which maintains genomic integrity through de novo synthesis of telomere repeats at chromosome ends. It is active in stem and germinal cells, thus sustaining physiological replication [1]. *TERT* is silenced in somatic cells where progressive telomere erosion, along with cell division, induces senescence and genetic alterations [1]. Congenital variations at the *TERT* coding sequence and of a number of genes involved in telomere biology are known in dyskeratosis congenita that is the prototype of telomere-related disorders, mainly affecting skin, lung, bone marrow (BM), and liver [2]. In cancer aberrant TERT expression contributes to immortalization through specific mechanisms inducing telomerase reactivation [1, 3]. This may occur by means of both methylation and mutations at *TERT* promoter (*TERT*_*P*_) [3]. In acute myeloid leukemia (AML), hypermethylation at THOR (*TERT* hypermethylated oncological region) was frequently found [4]. In solid tumors, somatic C > T hotspot transition at −124 and −146 nucleotides from the *TERT* ATG start site, and other rare mutations (−57A>C; −124/−125CC>TT; −138/−139CC>TT), are functionally activating by creating de novo binding sites for E-twenty-six (ETS) transcription factors [1]. Information about mutations in hematopoietic malignancies is scarce. To the best of our knowledge, *TERT*_*P*_ hotspot mutations have been described only in mantle cell lymphomas [5].

Here, we investigated *TERT*_*P*_ variants in a large series of myelodysplastic syndromes (MDS) and MDS/myeloproliferative neoplasms (MDS/MPN). Biological samples [cytogenetic preparations, genomic DNA, frozen and fresh BM, peripheral blood (PB) cells and nail cuttings] were obtained from patients referred to the Hematology Unit at the University of Perugia between 1995 and 2019. The study was conducted according to Helsinki declaration and approved by the Institutional Bioethics Committee (University of Perugia Protocol No. 2017-19R). Written informed consent was obtained from all patients and controls. New and rare (i.e., minor allele frequency <0.01) *TERT*_*P*_ variants were analyzed in silico through JASPAR Database and in vitro using Luciferase Reporter assay (Supplementary Table 1). *TERT*_*P*_-positive cases were further screened by Sanger Sequencing for the rs2853669 T>C single-nucleotide polymorphism, since it was previously shown to modulate mutated *TERT*_*P*_ *in* *cis* [6]. In addition, 30 myeloid leukemogenic genes and 35 telomere-related genes were investigated by next generation sequencing (NGS) using, respectively, the commercial Myeloid solution^TM^ and a Custom Hereditary Hematological Disorders gene panel provided by SOPHiA Genetics (Saint Sulpice, Switzerland). Telomere length (TL) was measured by Quantitative-Fluorescence In Situ Hybridization (Q-FISH) and/or Quantitative PCR (qPCR). For additional details, see Supplementary Methods, available on the Leukemia website.

We recruited 37 MDS/MPN and 350 MDS (250 males, 137 females; median age 74, range 7–94). Supplementary Table 2 shows demographics, hematological and cytogenetic features of all cases.

**Fig. 1:**
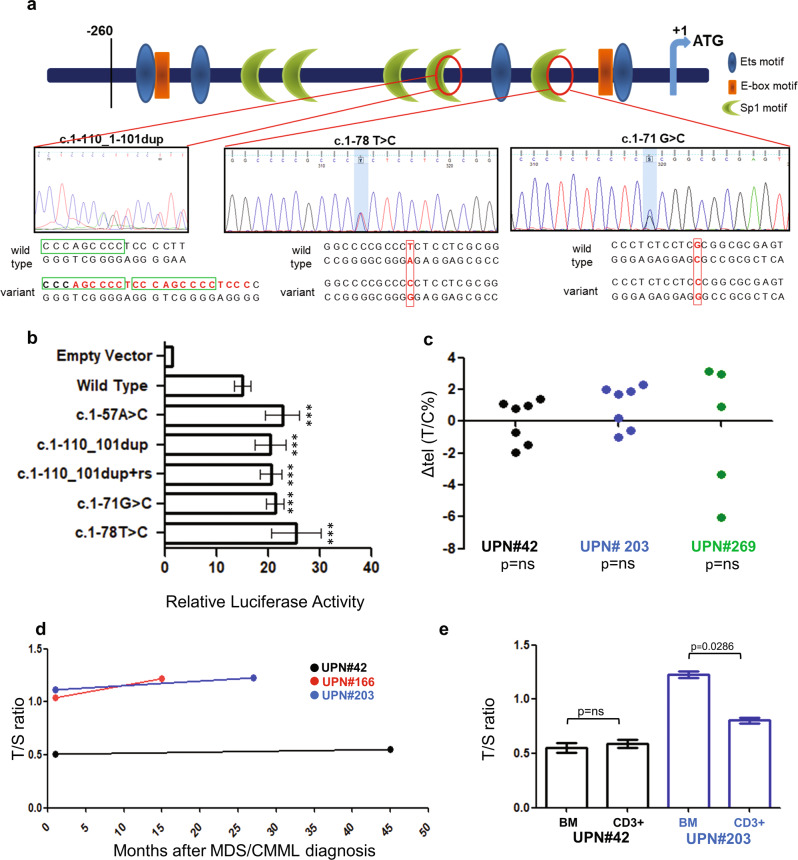
New and rare *TERT*_*P*_ activating variants increased promoter activity and TL investigations in malignant BM and PB cells from *TERT*_*P*_-positive cases did not reveal shortening as expected. **a** Schema of *TERT*_*P*_ with transcription factor binding sites (blue, orange, and green symbols) and new/rare *TERT*_*P*_ variants in myeloid malignancies (red circles). The c.1-110_1-101dup included duplication of an Sp1 binding site (green square), between nucleotides 1295095 and 1295102 (ENSG00000164362 GRCh38.p12). Adapted from Heidenreich and Kumar^1^ with permission. **b** Relative Luciferase activity in HeLa cell line; rs refers to rs2853669 T>C. Data are shown as mean ± SD in four independent experiments. The construct with the known activating c.1-57A>C was used as an internal positive control to validate data. ***p < 0.001 vs. wild type (two sample *t*-test with equal variances). **c** Inter-individual TL analysis by Q-FISH on PHA-stimulated PB metaphases. Unique patient number (UPN) refers to Supplementary Table 2. TL is expressed as T/C%. Each dot represents the difference (ΔTL T/C%) between mean TL in each patient and each age- and sex-matched healthy control (for UPN#42 and #203: 7 males, age range 71–83, mean 78.2, median 81, and for UPN#269: 5 females, age range 64–75, mean 72, median 74). ns, not significant (one sample Student’s *t* test). **d** Intra-individual TL over time according to qPCR on  BM DNA at diagnosis and during the disease course. TL is expressed as T/S ratio. **e** Intra-individual TL analysis by qPCR comparing BM DNA with PB CD3^+^ cells negative for somatic mutations. TL is expressed as T/S ratio. Data are shown as mean ± SD in four independent experiments (*p* < 0.05, Mann–Whitney *U* test).

Sanger sequencing revealed *TERT*_*P*_ variants in 6/387 cases (1.5%, Table 1), including five MDS and one chronic myelomonocytic leukemia (CMML). *TERT*_*P*_ variants affected the hotspot nucleotide at −124 base pairs upstream to the *TERT* ATG start site in three cases. Two of them carried the c.1-124C>T hotspot, while the third one had a C>A substitution (Table 1). Both of these variants were previously shown to significantly increase *TERT*_*P*_ activity [7]. Two more cases bore hitherto unknown variants, which were not found neither in our screening of PB from 200 healthy controls nor in dedicated databases, i.e., a c.1-110_1-101dup and a germline c.1-71G>C (Fig. 1a, Supplementary Table 3). The last case of this series carried a germline c.1-78T>C rs1467435130 variant (Fig. 1a, Table 1).

**Table 1:** Clinical, hematological, cytogenetic, and molecular data in *TERT*_*P*_*-*positive cases (UPN refers to Supplementary Table 2).

CASE (DNA tested)	Sex	Age	Diagnosis/IPSS-R/CPSS	Myeloid gene panel (VAF%)^a^	BM karyotype	*TERT*_*P*_ rs2853669 T>C	*TERT*_*P*_ (status, germline DNA tested)	Telomere gene PANEL^a^ (rs, VAF%), ACMG classification	Disease course (follow-up, months; death cause)
UPN #42 (BM)	M	77	MDS-RS-SLD/low	*SF3B1* c.2098 A>G, p.K700E (38.5%) *TET2* c.4957_4958del, p.S1653fs*6 (18.4%) *EZH2* c.2069 G>A; p.R690H (22.7%); c.437T>C, p.I146T (2.3%)	46,XY[16]	T/T	c.1-78T>C (rs1467435130) (germline, CD3^+^ PB cells)	neg	Stable (died, 57; sepsis)
UPN #166 (BM)	M	72	MDS-MLD/high	*EZH2* c.1505+3delG (33.6%)^b^ *ASXL1* c.2061T>A, p.C687* (35.2%) *ETV6* c.1228_1229delinsCC, p.E410P (27.4%) *CEBPA* c.1000G>A, p.E334K (28%)	47,XY,+8[3]/46,XY[16]	T/C	c.1-71G>C (germline, BM fibroblasts) *in* *cis* with rs2853669 T	*RECQL4* c.2561C>T, p.Thr854Ile (rs1167531855, 52%), LB *TERT* c.630C>T, p.A210= (rs1164854748, 50.9%), VUS	Evolution (died, 21)
UPN #199 (BM)	M	80	MDS-MLD/low	*ETV6* c.215G>A, p.W72* (1.6%) *DNMT3A*c.2082+3A>G (11.1%)^b^ *CSF3R* c.1358A>G, p.N453S (48.4%)^c^	46,XY[20]	C/C	c.1-110_1-101dupAGCCCCTCCC (na)* in* *cis* with rs2853669 C	*RIF1* c.5856T>C, p.Ser1952= (rs756019996, 48.5%), VUS *TERT* c.3107-4G>A (rs780229179, 46.8%) LB	Stable (died, 24; comorbidities)
UPN #203 (BM)	M	74	CMML-0^d^/low	*TET2* c.5659_5663dup^e^, p.Asn1888Lysfs*22 (92.9%)	46,XY[20]	T/T	c.1-124C>T^e^ (somatic, CD3^+^ PB cells, nail cuttings)	*TERF2* c.973A>G, p.Ile325Val (rs772066928, 49.6%), VUS	Stable (+58)
UPN #269 (BM)	F	72	MDS-SLD/low	*TET2* p.Asn1346Ilefs*17, c.4037del (24.6%)	46,XX[20]	T/T	c.1-124C>A (somatic, CD3^+^ PB cells, nail cuttings)	*DKC1* c.1497_1499dup, p.K500dup (rs797045523, 38.2%), B *RAD50* c.3113_3116del, p.Arg1038Asnfs*4 (52.5%), P	Stable (+48)
UPN #277 (BM)	F	55	MDS with isolated del(5q)/low	*MPL* c.1537_1539del, p.L513del (1.9%)	46,XX,del(5)(q13q31)[18]	T/C	c.1-124C>T (na) *in* *cis* with rs2853669 C	neg	Evolution (died, 138)

*UPN* unique patient number, *BM* bone marrow, *PB* peripheral blood, *VAF* variant allele frequency, *M* male, *F* female, *IPSS-R* revised international prognostic scoring system, *CPSS* chronic myelomonocytic leukemia-specific prognostic scoring system, *MDS-RS-SLD* myelodysplastic syndrome with ring sideroblasts and single lineage dysplasia, *MDS-MLD* myelodysplastic syndrome with multilineage dysplasia, *CMML-0* chronic myelomonocytic leukemia, type 0, *MDS-SLD* myelodysplastic syndrome with single lineage dysplasia, *na* not available data, *WT* wild type, *neg* negative, *ACMG* American College of Medical Genetics (see Supplementary Methods), *VUS* variant of uncertain significance, *B* benign variant, *LB* likely benign variant, *P* pathogenetic variant.

^a^See Supplementary Methods.

^b^Broken wild type splice site according to Human Splice Finder 3.1 (http://umd.be/HSF3/).

^c^Putative germline variant.

^d^Seventy-two months after diagnosis of diffuse large B-cell lymphoma (DLBCL) stage IIIB.

^e^Found at both DLBCL and CMML diagnosis.

Sequencing and in silico analysis (JASPAR Database) showed the *TERT*_*P*_ c.1-110_1-101dup produced duplication of binding sites for Sp1, a member of Specificity Protein/Krüppel-Like Factor transcription factor family (Fig. 1a and Supplementary Table 4) that, similarly to ETS family, was associated with *TERT*_*P*_ activation [1]. A significantly increased binding of transcription factors belonging to the same families was also generated by the two germline variants (c.1-71G>C and c.1-78T>C, Supplementary Table 4).

In two families with the same c.1-57A>C variant and early onset melanoma, a pathogenetic role in cancer development was suggested for the germline *TERT*_*P*_ variant [1]. In the family bearing the c.1-78T>C substitution, however, neither blood nor solid tumors emerged in six carriers identified across three generations (age range 18–73 years, mean age 47.5, median 52), suggesting that our variant “*per se*” is not promoting malignancy (Supplementary Fig. 1).

According to in silico analysis, the Luciferase Reporter assay showed that all three variants caused a significantly increased *TERT*_*P*_ activity by 1.3–1.7 fold in HeLa cells (Fig. 1b). Notably, adding evidence for a cell context-dependent action [6], the rs2853669 polymorphic C allele had no effect against our c.1-110_1-101dup (Fig. 1b).

As recurrent *TERT*_*P*_ mutations have been previously found in individuals with telomerase deficiency due to pathogenetic variants at the coding sequence of *TERT*, *TERC* and *PARN* [8, 9], we tested these genes and extended our NGS analysis to 32 additional telomere-related genes. Results showed six heterozygous variants classified as benign, likely benign, or of uncertain significance, according to the American College of Medical Genetics criteria (Table 1, Supplementary Methods). Among them there were two synonymous (*TERT*, *RIF*), two missense (*RECQL4*, *TERF2*), one splice site (*TERT*) variant and one inframe duplication (*DKC1*). In addition, we found a pathogenetic frameshift heterozygous variant at *RAD50* (Table 1). Although the significance of this observation remains to be clarified, deleterious *RAD50* variants in heterozygosity have never been reported in a telomere-related phenotype [10]. Based on all these results, we excluded a congenital defect of the telomerase underlying *TERT*_*P*_ variants in this series.

We further extended our investigations to dysplastic BM cells by both conventional cytogenetics and a myeloid NGS panel. Acquired cytogenetic aberrations and/or mutations were identified in all cases, suggesting cooperation between *TERT*_*P*_ and disease-related somatic hits (Table 1). Similarly, *TERT*_*P*_ mutations were previously reported in bladder cancer with *FGFR3* mutations and in both thyroid cancer and melanoma with *BRAF* mutations, favoring the hypothesis that telomerase reactivation supports the proliferation of oncogene-transformed cells [1]. However, since telomerase is constitutively active in hematopoietic stem cells, the significance of its activation in myeloid malignancies is less clear [1].

Predominant somatic events in our cases were loss of function mutations at one or more epigenetic genes, namely, *TET2* and *EZH2*. Interestingly in murine embryonic stem cells, *TET2* deficiency led to sub-telomeric hypermethylation and telomere shortening via a telomerase-independent effect [11]. In human glioma cell lines, *EZH2* depletion reduced TERT level [12]. Its inhibition in a human osteosarcoma cell line decreased telomeric heterochromatic marks [13]. Furthermore, in human AML cell lines, DNA methyltransferases inhibition by 5-azacytidine induced DNA damage at telomeres, telomere shortening, and downregulation of TERT expression [14]. Altogether these data suggest a functional link between epigenetic genes and *TERT*_*P*_ variants, that in our series might have counterbalanced acquired telomere disturbances. Although this hypothesis remains to be proved, insights were generated from studies on TL. First, in three *TERT*_*P*_ cases positive for *TET2* (and also for *EZH2* in one out of three) the TL in PB was not reduced, compared with age- and sex-matched healthy controls (Fig. 1c). This was rather unexpected as telomere shortening is typically found in MDS [15]. Moreover, in intra-individual longitudinal studies TL in BM cells was almost stable over time (Fig. 1d) and it was similar or even slightly longer than that of CD3^+^ lymphocytes without epigenetic mutations (Fig. 1e, Supplementary Fig. 2). Notably, in MDS telomere attrition was shown to preferentially affect the myeloid versus the lymphoid compartment [16].

In conclusion, our study for the first time identified somatic and germline activating *TERT*_*P*_ variants in both MDS and CMML and provided insights on their close association with acquired mutations at epigenetic modulators. Inclusion of *TERT*_*P*_ screening in the diagnostic routine of MDS and MDS/MPN will be helpful to better assess the biological and clinical significance of *TERT*_*P*_ variants.

## Supplementary information

Revised Supplemental document
