# Supplementary material for: Activating somatic and germline TERT promoter variants in myeloid malignancies
Source: Leukemia. 2020 May 4;35(1):274–8. doi: 10.1038/s41375-020-0837-6 (PMC7787968; doi:10.1038/s41375-020-0837-6)
Supplement: Supplementary file 1 — Revised Supplemental document [file 41375_2020_837_MOESM1_ESM.doc]

**Supplement to: Activating Somatic and Germline *TERT* Promoter Variants in Myeloid Neoplasms**

Valeria Nofrini*, Caterina Matteucci*, Fabrizia Pellanera, Paolo Gorello, Danika Di Giacomo, Anair Graciela Lema Fernandez, Carlotta Nardelli, Tamara Iannotti, Lucia Brandimarte, Silvia Arniani, Martina Moretti, Alessio Gili, Giovanni Roti, Valeria Di Battista, Simona Colla, Cristina Mecucci

*These authors contributed equally to the study

**Table of contents**

[Supplementary Methods](#__RefHeading___Toc15307) 1

Supplementary Table 1. Primers used for PCR amplification in Luciferase Reporter Assay  [6](#__RefHeading___Toc15308)

Supplementary Table 2. Demographic, hematological and cytogenetic features of all patients analyzed for TERTP……………………………………………………………………7

[Supplementary Table 3. List of consulted population, disease-specific and sequence databases 19](#__RefHeading___Toc15309)

[Supplementary Table 4. Bioinformatic analyses of sequences surrounding the *TERT_P_* new/rare variants using JASPAR Database 20](#__RefHeading___Toc15310)

[Supplementary Figure 1. Family tree of patient UPN#42 carrying *TERT_P_* c.1-78T>C variant 22](#__RefHeading___Toc15312)

[Supplementary Figure 2. Intra-individual TL in patient UPN#203 (Supplementary Table 2) as analysed by Q-FISH on unstimulated BM cells and PHA-stimulated PB metaphases .. 2](#__RefHeading___Toc15308)3

**Supplementary Methods**

**Cytogenetics and mutational analysis**

Karyotypes were obtained after 24h cultures and G-banding with Wright stain. Cytogenetic abnormalities were described using the 2016 International System for Human Cytogenetic Nomenclature [1].

Mutational analysis for *TERT* core promoter (*TERT_P_*) was performed by Denaturing High Performance Liquid Chromatography (DHPLC, Wave® MD system; ADS BIOTEC Inc. Omaha, NE) and/or Sanger sequencing (ABI 3500 Genetic Analyzer, Applied Biosystem) with the appropriate forward (5'-GTCCTGCCCCTTCACCTTC-3') and reverse (*5'-AGCACCTCGCGGTAGTGG-3)* primers. Purified peripheral blood (PB) CD3^+^ T-lymphocytes, nail cuttings or bone marrow (BM) fibroblasts were used as germline counterparts.

Positive cases underwent: *(i)* screening for *TERT_P_* rs2853669 single nucleotide polymorphism (SNP) (forward primer 5'-GATTCGACCTCTCTCCGCTG-3'; reverse primer *5'-CACCAGCGCGCGGAAAGC-3'); (ii)* in depth genetic characterization by next generation sequencing (NGS). Two NGS panel provided by SOPHiA Genetics (Saint Sulpice, Switzerland) were used. The Myeloid Solution^TM^ investigated 30 genes involved in myeloid leukemogenesis i.e *ABL1* (exons 4-9), *ASXL1* (9,11,12,14), *BRAF* (15), *CALR* (9), *CBL* (8,9), *CEBPA* (all), *CSF3R* (all), *DNMT3A* (all), *ETV6* (all), *EZH2* (all), *FLT3* (13-15,20), *HRAS* (2,3), *IDH1* (4), *IDH2* (4), *JAK2* (all), *KIT* (2,8-11,13,17,18), *KRAS* (2,3), *MPL* (10), *NPM1* (10,11), *NRAS* (2,3), *PTPN11* (3,7-13), *RUNX1* (all), *SETBP1* (4), *SF3B1* (10-16), *SRSF2* (1), *TET2* (all), *TP53* (2-11), *U2AF1* (2,6), *WT1* (6-10), *ZRSR2* (all), and the Custom Hereditary Hematological Disorders (CHHD_A_v1) gene panel analyzed 13 genes known to be involved in Telomere Biology Disorders pathogenesis [2], reported in bold, as well as 22 additional genes connected with telomere biology (*ASF1a*, *ASF1b*, *ATRX*, BLM, ***CTC1***, *CTCF*, *DAXX*, *DCLRE1B*, ***DKC1***, *FUS*, *GAR1*, *MRE11*, *NBS1*, PABPN1, ***NOP10***, ***NHP2***, ***NAF1***, ***PARN****, PIF1*, *PINX1*, ***POT1***, *RAD50*, *RECQL4*, *RIF1*, ***RTEL1***, ***STN1****,* ***TERC****, TEN1*, *TERF1, TERF2, TERF2IP,* ***TERT****,* ***TINF2****, USB1,* ***WRAP53***).

Libraries were prepared using 200 ng of BM genomic DNA following manufacturer’s instructions (SOPHiA Genetics, Saint Sulpice, Switzerland). Pooled libraries were sequenced on MiSeq Reagent kit v.2 (Tumour Solution^TM^) and v.3 (CHHD_A_v1 custom solution) on an Illumina MiSeq Sequencer (Illumina, San Diego, CA). Fastq files were analyzed with Sophia DDM software (version 5.3.9.2, SOPHiA Genetics, Saint Sulpice, Switzerland). Exonic and splice site variants were taken into consideration and filtered considering those with a minor allele frequency (MAF) <0.01. Variants emerging from CHHD_A_v1 NGS panel were further filtered retaining those with a variant allele frequency >20% and with read depth >500 to isolate germline events which were classified according to the American College of Medical Genetics guidelines [3].

***In silico* analysis**

By means of the JASPAR Database transcription factor prediction tool [4], wild-type and variant sequences were compared and scanned for the *Homo sapiens* JASPAR CORE profiles, using the SCAN tool in JASPAR database Web server. When compared with the reference sequence, all variant profile scores ≥0.05 point were predicted to have significantly higher probability of binding [5]. Scores <0.05 point were predicted to have a significantly lower probability of binding [5].

**Luciferase Reporter Assay**

PCR products obtained using forward and reverse primers containing the BglII and HindIII cleavage sites, respectively (Supplementary Table 1), were first cloned into pGEM T-Easy vector (Promega, Madison, WI, USA), digested with BglII and HindIII and sub-cloned into the pGL4.10[luc2] vector (Promega, Madison, WI, USA), upstream of the Firefly luciferase gene .

HeLa cells [6] were maintained in Eagle's Minimum Essential Medium supplemented with 10% fetal bovine serum and antibiotics and incubated at 37°C and 5% CO_2_. They were seeded into 6-well plates (5X10^5^ cells/well) and the next day were co-transfected (Viafect Transfection Reagent, Promega), with 500ng of the reporter plasmid and 10:1 pGL4.74[hRluc/TK]. Twenty-four hours later lysed cells were analyzed for Firefly and Renilla luciferase activity using the Dual Glo Luciferase Assay System (Promega) according to the manufacturer’s instructions. Relative luciferase activity was normalized to Renilla luciferase activity in 4 independent experiments, each performed in triplicate.

**Telomere Length Measurement**

Telomere Length (TL) was measured at diagnosis and/or during monitoring by Quantitative-Fluorescence In Situ Hybridization (Q-FISH) and Quantitative PCR (qPCR) in two sets of experiments: *(i)* inter-individual analysis comparing each patient with 5-7 age and sex-matched normal controls; *(ii)* intra-individual analysis comparing the patient’s BM and purified CD3^+^ PB lymphocytes.

***Q-FISH*** was applied on metaphases obtained from PHA-stimulated PB T cells after 72 hours’ culture and from unstimulated BM cells after 24 hours’ culture, as previously described [7]. Q-FISH used a Cy3-linked telomeric PNA probe (PANAGENE) and a chromosome 2 centromeric PNA probe (DAKO). Analysis was performed with ISIS software (MetaSystems, Germany). TL was expressed as T/C% [8]. At least 25 metaphases were scored in each experiment. To ensure data reproducibility at least two independent experiments were performed for each patient.

***qPCR****.* TL was measured in genomic DNA from whole BM and purified PB CD3^+^ T-lymphocytes which was obtained through standard desalting procedures and stored at -20°C, using the monochrome multiplex quantitative PCR method, as previously described, with slight modifications [9,10]. Briefly, all pipetting steps were performed using a Qiagility pipettor (Qiagen). PCR was performed in a final volume of 20 μl containing 20 ng DNA, 1x PowerUp SYBRGreen Master Mix (Applied Biosystems), 0.1μM forward (5'-CGGTTTGTTTGGGTTTGGGTTTGGGTTTGGGTTTGGGTT-3') and 0.9μM reverse (*5'-GGCTTGCCTTACCCTTACCCTTACCCTTACCCTTACCCT -3')* telomere primers or 0.5μM forward (5'-CAGCAAGTGGGAAGGTGTAATCC-3')/reverse(*5'-CCCATTCTATCATCAACGGGTACAA-3')* *36B4* single copy gene primers. All primers were HPLC purified. PCR conditions were: 50°C 2 min, 95°C 2 min, then 40 cycles of denaturation (95°C, 15 sec), anneal/extension (54°C 1 minute) for telomeric DNA amplification (T); annealing (58°C), extension (72°C 1 min) for *36B4* amplification (S). For each sample T and S reactions were quantified, using absolute quantification, against a six-point standard curve, obtained from 4 pooled DNA samples prepared by serial dilutions (4 to 0.04096 ng/μL). Analysis was performed with LightCycler 480 II software (Roche Diagnostics, Mannheim, Germany) using the second-derivative maximum method and high-sensitivity detection algorithm, as previously reported [10]. The T/S ratio for each sample was normalized to the average T/S ratio of five replicates of an internal quality control calibrator sample, within the same plate, to yield the final standardized T/S ratio. For quality control all T and S reactions were measured in triplicate and Ct values were averaged if they met a percentage coefficient of variation (CV%) threshold <2%. Plate results were accepted if R^2^ of both telomere and 36B4 standard curves were >0.995 and intra-assay CV% of five replicate QC sample T/S ratios was <5%. To ensure data reproducibility each sample was assayed in at least three independent runs; the mean inter-assay CV% of standardized T/S ratios was 3.5%.

**Statistical analysis**

Statistical analysis was performed with GraphPad Prism software 5.01. For luciferase assay mutated vs wild-type constructs were tested using the two sample t-test with equal variances (α≤0.0001), the two sample Kolmogorov-Smirnov test for equal distribution of functions and the Pearson chi-square test (Fig. 1b).

For TL studies one sample Student's *t* test assessed the significance of differences in mean TL in each case *vs* healthy controls (Fig. 1c). The Mann-Whitney U test determined the significance of difference in mean TL between BM and CD3^+^ PB cells (Fig. 1e, Supplementary Fig. 2).

**Data sharing statement**

All original data and protocols are available through contacting the corresponding author (cristina.mecucci@unipg.it).

**References**

1. ISCN 2016. In: An international system for human cytogenomic nomenclature (2016) McGowan-Jordan J, Simons A, Schmid M, editors. Basel, Freiburg: Karger; 2016.

2. Niewisch MR, Savage SA. An update on the biology and management of dyskeratosis congenita and related telomere biology disorders. Expert Rev Hematol. 2019; **12**: 1037-52.

3. Nykamp K, Anderson M, Powers M, Garcia J, Herrera B, Ho YY, *et al*. Sherloc: a comprehensive refinement of the ACMG-AMP variant classification criteria. *Genet Med*. 2017; **19**: 1105-1117.

4. Khan A, Fornes O, Stigliani A, Gheorghe M, Castro-Mondragon JA, van der Lee R, *et al*. JASPAR 2018: update of the open-access database of transcription factor binding profiles and its web framework. *Nucleic Acids Res*. 2018;46(D1):D260-D266.

5. Allory Y, Beukers W, Sagrera A, Flández M, Marqués M, Márquez M *et al*. Telomerase reverse transcriptase promoter mutations in bladder cancer: high frequency across stages, detection in urine, and lack of association with outcome. *Eur Urol*. 2014; **65**: 360-366.

6. Takakura M, Kyo S, Kanaya T, Hirano H, Takeda J, Yutsudo M, *et al*. Cloning of human telomerase catalytic subunit (hTERT) gene promoter and identification of proximal core promoter sequences essential for transcriptional activation in immortalized and cancer cells. *Cancer Res*. 1999; **59**: 551-557.

7. Berardinelli F, Antoccia A, Cherubini R, De Nadal V, Gerardi S, Tanzarella C *et al.* Telomere alterations and genomic instability in long-term cultures of normal human fibroblasts irradiated with X rays and protons. *Radiat Prot Dosimetry*. 2011; **143**: 274-278.

8. Perner S, Brüderlein S, Hasel C, Waibel I, Holdenried A, Ciloglu N *et al.* Quantifying telomere lengths of human individual chromosome arms by centromere-calibrated fluorescence in situ hybridization and digital imaging. *Am J Pathol*. 2003; **163**: 1751-1756.

9. Cawthon RM. Telomere measurement by quantitative PCR. *Nucleic Acids Res*. 2002;30(10):e47.

10. Dagnall CL, Hicks B, Teshome K, Hutchinson AA, Gadalla SM, Khincha PP, *et al*. Effect of pre-analytic variables on the reproducibility of qPCR relative telomere length measurement. *PLoS One* 2017; **12**: e0184098.

**Supplementary Table 1. Primers used for PCR amplification in Luciferase Reporter Assay.**

| **Primers** | **Sequence (5’-3’)** | **Position(bp)** |
| --- | --- | --- |
|  |  | **from ATG start site** |
| *TERT*_ BglII_Forward_WT | AGATCT*ttt*CCAGGACCGCGCT**T**CCCAC | -200 to +73 |
| *TERT*_HindIII_Reverse | AAGCTT*t*GGGAGCGCGCGGCATC |  |
| *TERT*_ BglII_Forward_POL | AGATCT*ttt*CCAGGACCGCGCT**C**CCCAC | -200 to +73 with rs2853669 T>C SNP |
| *TERT*_HindIII_Reverse | AAGCTT*t*GGGAGCGCGCGGCATC |  |

Underlines indicate restriction enzyme site for BglII and HindIII. *In italics*: one or two thymidines added between enzyme cleavage site and primer sequence. **In bold**: site specific mutagenesis introduced for rs2853669 SNP. WT, wild-type; POL, polymorphic.

**Supplementary Table 2. Demographic, hematological and cytogenetic features of all patients analyzed for *TERT_P_*.**

| **PATIENT NUMBER** | **SEX** | **AGE** | **DIAGNOSIS** | **BONE MARROW KARYOTYPE** |
| --- | --- | --- | --- | --- |
| UPN #1 | F | 81 | MDS-EB-1 | 46,XX |
| UPN #2 | M | 81 | MDS-MLD | 46,XY |
| UPN #3 | F | 57 | MDS-EB-1 | 46,XX |
| UPN #4 | M | 73 | MDS-EB-1 | 46,XY |
| UPN #5 | F | 77 | MDS-EB-1 | 46,XX |
| UPN #6 | F | 74 | MDS-EB-1 | 46,XX |
| UPN #7 | F | 60 | MDS-EB-1 | 46,XX |
| UPN #8 | F | 78 | MDS-EB-1 | 46,XX |
| UPN #9 | M | n.a. | MDS-EB-1 | 46,XY |
| UPN #10 | M | 81 | MDS-EB-1 | 46,XY |
| UPN #11 | M | 88 | MDS-EB-2 | 46,XY |
| UPN #12 | M | 47 | MDS-EB-2 | 46,XY |
| UPN #13 | M | 62 | MDS-EB-2 | 46,XY |
| UPN #14 | M | 80 | MDS-EB-1 | 46,XY |
| UPN #15 | M | 78 | MDS-EB-1 | 46,XY |
| UPN #16 | M | 74 | MDS-EB-2 | 46,XY |
| UPN #17 | M | 80 | MDS-MLD | 46,XY |
| UPN #18 | F | 72 | MDS-SLD | 46,XX |
| UPN #19 | M | 74 | MDS-SLD | 46,XY |
| UPN #20 | F | 70 | MDS-MLD | 46,XX |
| UPN #21 | F | 80 | MDS-SLD | 46,XX |
| UPN #22 | M | 65 | MDS-MLD | 46,XY |
| UPN #23 | M | 62 | MDS-RS-SLD | 46,XY |
| UPN #24 | F | 74 | MDS-MLD | 46,XX |
| UPN #25 | M | 87 | MDS-MLD | 46,XY |
| UPN #26 | M | 76 | MDS-MLD | 46,XY |
| UPN #27 | M | 80 | MDS-MLD | 46,XY |
| UPN #28 | M | 82 | MDS-MLD | 46,XY |
| UPN #29 | M | 77 | MDS-MLD | 46,XY |
| UPN #30 | F | 83 | MDS-MLD | 46,XX |
| UPN #31 | M | 85 | MDS-MLD | 46,XY |
| UPN #32 | M | 71 | MDS-MLD | 46,XY |
| UPN #33 | F | 79 | MDS-MLD | n.a. |
| UPN #34 | M | 81 | MDS-MLD | 46,XY |
| UPN #35 | M | 88 | MDS-MLD | 46,XY |
| UPN #36 | F | 60 | MDS-MLD | 46,XY |
| UPN #37 | M | 81 | MDS-MLD | 46,XY |
| UPN #38 | M | 67 | MDS-MLD | 46,XY |
| UPN #39 | M | 94 | MDS-MLD | 46,XY |
| UPN #40 | M | 39 | MDS-MLD | 46,XY |
| UPN #41 | F | 65 | MDS-MLD | 46,XX |
| UPN #42 | M | 77 | MDS-RS-SLD | 46,XY |
| UPN #43 | F | 54 | MDS-MLD | 46,XX |
| UPN #44 | M | 56 | MDS-SLD | 46,XY |
| UPN #45 | F | 70 | MDS-RS-SLD | 41-45,XX,-22/46,XX |
| UPN #46 | F | 76 | MDS-MLD | 46,XX |
| UPN #47 | M | 51 | MDS-EB-1 | 45,XY,del(5)(q13q31),-7,del(20)(q11q13)/46,XY |
| UPN #48 | F | 81 | MDS-EB-1 | 44,XX,-3,der(5)(del(5)(q13q34)add(5q34),-6,-7,der(7)t(3;7)(q21;q36),del(11)(q11),der(16)t(7;16)(q21;q24), -17,+mar1, + mar2 |
| UPN #49 | M | 49 | MDS-EB-2 | 45,XY,-5,-7,del(12)(p13),del(13)(q21),del(20)(q11q13),+mar/46,XY |
| UPN #50 | M | 44 | MDS-EB | 43-45,XY,del(1)(q32),del(5)(q31q35),del(6)(q?),-15,der(17)(?),-22,+mar/46,XY |
| UPN #51 | F | 62 | MDS-EB-1 | 46,XX,del(5)(q13q33),del(7)(q22q32),del(7)(q22q32),der(20)/46,XX |
| UPN #52 | M | 63 | MDS-RS-SLD | 40-48,XY,t(1;3)(p32;p21),-5,-7,-13,-18,-20,-22,+ mar/46,XY |
| UPN #53 | M | 80 | MDS-MLD | 45,XY,del(5)(q13,q31),del(7q), ?der(12)dic(3;12)(p12;p12)/46,XY |
| UPN #54 | F | 64 | MDS-MLD | 46,XX/41-45,XX,dic(2;11)(p11;p11),-4,-7,del(7)(q31q36),+1,-3mar |
| UPN #55 | F | 78 | MDS-MLD | 45,XX,idem,-11,add(13)(p11),+mar/45,XX,-3,del(5)(q?)/46,XX |
| UPN #56 | M | 75 | MDS-MLD | 45,XY,-4,add(5)(q21),del(6)(q12),add(19)(p13),-21,+mar |
| UPN #57 | M | 78 | MDS-U | 46,XY,-5,del(7)(q21),+mar/46,XY |
| UPN #58 | M | 65 | MDS-MLD | 46,XY,del(4)(q?),del(5)(q13q34),-7,+mar/46,XY |
| UPN #59 | F | 81 | MDS-MLD | 47,XX,-3,-5,-11,+13,add(7q),+mar |
| UPN #60 | M | 61 | MDS-U | 49,XY,+1,del(5)(q13q31),+9,+11,-13,-17,+19,-22,+mar1,+mar2 |
| UPN #61 | M | 52 | MDS-U | 45,XY,del(5)(q13q34),-7,del(12)(p13),-18,-20,+mar1,+mar2 |
| UPN #62 | M | 66 | MDS-U | 43-45,XY,t(3;6)(p21;q27),-5,-7,-12,-22,+mar1,+mar2 |
| UPN #63 | F | 68 | MDS-EB | 47,XX,del(5)(q13q33),+8/37-45,XX,hsr(4q),+8,add(14)(p11),add(16)(p13),-17,-19,+1-5mar/46,XX |
| UPN #64 | M | 73 | MDS-EB-2 | 45,X,der(11)t(11;18)(q13;q11)/44,X,-Y,der(11)t(11;18)(?;?),-18 |
| UPN #65 | M | 67 | MDS-RS-SLD | 46,X,-Y,t(1;19;13)(p13;p13;q11),del(5)(q13q34)/48-49,idem,+mar1,+mar2 or 2xmar2 |
| UPN #66 | F | n.a. | MDS-EB | 44,XX,del(3)(q),del(5)(q),-7,del(17)(p),-21/43,idem,-20/44,XX,idem,dic(12;20)(p11-12;q21q22)+mar |
| UPN #67 | M | 67 | MDS-EB-1 | 48,idem,+6,+8,del(12p)/46,XY,del(20)(q11q13)/46,XY |
| UPN #68 | F | n.a. | MDS-EB-2 | 45,XX,-2,-4,-7,-11,+3mar |
| UPN #69 | F | 73 | MDS-EB-2 | 45-48,XX,der(2)t(2;4)(p23,q?),+der(2)t(2;?)(p?;?),der(3)t(3;?)(p21;?),-4,-5,-7,+8,der(12),+18,+mar |
| UPN #70 | F | 83 | MDS-EB-1 | 46,XX,del(5)(q31q35)/46,XX |
| UPN #71 | F | 77 | MDS-EB-2 | 46,XX,del(5q)(q13q31)/46,XX |
| UPN #72 | M | 72 | MDS-EB | 46,XY,del(5)(q13q33) |
| UPN #73 | M | 73 | MDS with isolated del(5q) | 46,XY,del(5q)(q13q31) |
| UPN #74 | F | 68 | MDS with isolated del(5q) | 46,XX,del(5)(q13q35)/ 46,XX |
| UPN #75 | F | 77 | MDS-SLD | 46,XX,del(5)(q13q31)/ 46,XX |
| UPN #76 | F | 46 | MDS with isolated del(5q) | 47,XX,del(5)(q13q31),+21/46,XX |
| UPN #77 | M | 52 | MDS-EB-1 | 46,XY,t(2;11)(p21;q24),del(5q) (q13q34) |
| UPN #78 | M | 71 | MDS-EB-1 | 46,XY/45,XY,del(5q)(q13q34),-18 |
| UPN #79 | F | 51 | MDS-EB-1 | 46,XX,del(20)(q11q13)/46,XX |
| UPN #80 | M | 58 | MDS-EB-2 | 46,XY,del(20)(q11q13)/46,XY |
| UPN #81 | M | 79 | MDS-EB-2 | 46,XY,del(20)(q11q12)/46,XY |
| UPN #82 | M | 73 | MDS-SLD | 46,XY,del(20)(q11q13)/46,XY |
| UPN #83 | M | 65 | MDS-RS-SLD | 46,XY,del(20q)(q11q13)/ 47,idem,+8/ 46,XY |
| UPN #84 | M | 73 | MDS-MLD | 46,XY,del(20)(q11q13)/46,XY |
| UPN #85 | F | 61 | MDS-MLD | 46,XX,del(20)(q11q13)/ 46,XX |
| UPN #86 | M | 78 | MDS-MLD | 46,XY,del(20)(q11q13) |
| UPN #87 | M | 86 | MDS-MLD | 46,XY,del(20)(q11q13) 46,XY |
| UPN #88 | M | 66 | MDS-MLD | 46,XY,del(20)(q11q13)/47,XY,+12/46,XY |
| UPN #89 | M | 86 | MDS-U | 46,XY,del(20)(q11q13) |
| UPN #90 | F | 82 | MDS-EB-1 | 46,XX,del(20)(q11q13)/46,XX |
| UPN #91 | M | 91 | MDS-MLD | 46,XY,del(20)(q11q13)/46,XY |
| UPN #92 | F | 28 | MDS-EB-1 | 46,XX,t(3;3)(q21;q26) |
| UPN #93 | F | 52 | MDS-EB-2 | 47,XX,+21/46,XX |
| UPN #94 | M | 79 | MDS-EB-2 | 46,XY,i(17)(q10)/47,idem,+19/46,XY |
| UPN #95 | M | 82 | MDS-EB | 46,XY,del(5)(q?),der(17)(p13)/46,XY |
| UPN #96 | F | 75 | MDS-EB | 47,XX,+8/46,XX |
| UPN #97 | M | 70 | MDS-EB | 46,XY,del(11)(q13q23)/46,XY |
| UPN #98 | M | 7 | MDS-MLD | 45,XY,-7 |
| UPN #99 | M | 66 | MDS-MLD | 45,X,-Y/46,XY |
| UPN #100 | M | 78 | MDS-MLD | 47,XY,+8/46,XY |
| UPN #101 | M | 76 | MDS-MLD | 45,X,-Y/46,XY |
| UPN #102 | F | 23 | MDS-MLD | 46,XX |
| UPN #103 | F | 69 | MDS-MLD | 46,XX,t(1;12)(q25;p13)/46,XX |
| UPN #104 | F | 73 | MDS-RS-SLD | 46,XX,del(11)(q14q25)/46,XX |
| UPN #105 | M | 60 | MDS-U | 45,X,-Y/46,XY |
| UPN #106 | M | 65 | MDS-EB-1 | 46,XY |
| UPN #107 | M | 63 | MDS-MLD | 46,XY |
| UPN #108 | F | 64 | MDS-EB-2 | 46,XX, t(X;6)(p11;q13)/46,XX |
| UPN #109 | F | 82 | MDS-MLD | 46,XX/46,XX,del(1)(p21p31),del(11)(q?), -12,+mar |
| UPN #110 | M | 70 | MDS-MLD | 46,XY,del(20)(q)/45,XY,-7,del(20)(q) |
| UPN #111 | F | 69 | MDS-EB-1 | 46,XX |
| UPN #112 | M | 86 | MDS-EB-2 | 45,X,-Y/46,XY |
| UPN #113 | M | 73 | MDS-MLD | 47,XY+13/46,XY |
| UPN #114 | F | 52 | MDS-EB-2 | 46,XX |
| UPN #115 | M | 70 | MDS with isolated del(5q) | 46,XY,del(5)(q13q33) |
| UPN #116 | F | 82 | MDS-MLD | 46,XX,t(1;3)(p36.2;q21) |
| UPN #117 | F | 46 | MDS-EB-2 | 46,XX,del(5)(q13q33)/46,XX |
| UPN #118 | M | 53 | MDS-SLD | 46,XY |
| UPN #119 | M | 76 | MDS/MPN-U | 46,XY |
| UPN #120 | F | 68 | MDS/MPN-U | 46,XX |
| UPN #121 | M | 67 | MDS/MPN-U | 46,XY |
| UPN #122 | M | 67 | MDS-MLD | 46,XY |
| UPN #123 | M | 70 | MDS-EB-1 | 46,XY |
| UPN #124 | F | 78 | MDS/MPN-U | 46,XX |
| UPN #125 | M | 78 | MDS-U | 46,XY |
| UPN #126 | M | 71 | MDS-EB-2 | 46,XY |
| UPN #127 | M | 83 | MDS-EB-1 | 46,XY |
| UPN #128 | M | 75 | MDS-SLD | 46,XY |
| UPN #129 | F | 77 | MDS-MLD | 46,XX,del(5)(q31q35) |
| UPN #130 | F | 76 | MDS-MLD | 46,XX |
| UPN #131 | M | 59 | MDS-MLD | 46,XY |
| UPN #132 | M | 84 | MDS-SLD | 46,XY |
| UPN #133 | F | 74 | MDS-EB-2 | 46,XX |
| UPN #134 | F | 80 | MDS-U | 47,XX,+8/46,XX |
| UPN #135 | F | 79 | MDS-MLD | 46,XX |
| UPN #136 | M | 75 | MDS-MLD | 46,XY,+1, dic(1;7)(p11;q11)/46,XY |
| UPN #137 | M | 81 | MDS-EB-1 | 46,XY |
| UPN #138 | M | 79 | MDS-MLD | 46,XY |
| UPN #139 | M | 88 | MDS-RS-SLD | 45,X,-Y/46,XY |
| UPN #140 | M | 85 | MDS-MLD | 46,XY,del(20)(q11q13)/47,idem,+del(20)(q11q13)/46,XY |
| UPN #141 | F | 81 | MDS with isolated del(5q) | 46,XX,del(5)(q)(q13q33)/46,XX, |
| UPN #142 | M | 77 | MDS-EB-1 | 43-45;XY,-5,del(7)(q21q36),-9,add(12)(p11),-13,+16,add(17)(p11),+19,del(21)(q?) |
| UPN #143 | M | 73 | MDS-MLD | 46,XY |
| UPN #144 | M | 76 | MDS-MLD | 46,XY |
| UPN #145 | M | 75 | MDS-MLD | 46,XY |
| UPN #146 | F | 59 | MDS-MLD | 47XX,+11/46,XX |
| UPN #147 | M | 77 | MDS-SLD | 46,XY |
| UPN #148 | F | 74 | MDS-SLD | n.a. |
| UPN #149 | M | 91 | MDS-MLD | 45,X,-Y/46,XY |
| UPN #150 | F | 89 | MDS-MLD | n.a. |
| UPN #151 | M | 73 | MDS-SLD | 46,XY |
| UPN #152 | F | 82 | MDS-MLD | 46,XX |
| UPN #153 | F | 78 | MDS-MLD | 46,XX |
| UPN #154 | M | 61 | MDS-MLD | 46,XY |
| UPN #155 | F | 73 | MDS-MLD | 46,XX |
| UPN #156 | M | 82 | MDS-EB-1 | 46,XY |
| UPN #157 | M | 57 | MDS-EB-2 | 46,Y,add(X)(p21),-11,+ring/46,XY/46,idem,del(5)(q13q31) |
| UPN #158 | F | 80 | MDS-MLD | 46,XX |
| UPN #159 | M | 90 | MDS-SLD | 46,XY |
| UPN #160 | M | 58 | MDS-MLD | 46,XY |
| UPN #161 | M | 82 | MDS-EB-1 | 47,XX,+8,del(11)(q23) |
| UPN #162 | F | 76 | MDS-MLD | 46,XX |
| UPN #163 | F | 72 | MDS-MLD | 46,XX |
| UPN #164 | F | 84 | MDS-RS-SLD | 46,XX |
| UPN #165 | F | 86 | MDS-MLD | 46,XX,del(20)(q11q13) |
| UPN #166 | M | 72 | MDS-MLD | 47,XY,+8/46,XY |
| UPN #167 | F | 75 | MDS with isolated del(5q) | 46,XX,del5(q13q31) |
| UPN #168 | M | 66 | MDS-MLD | 46,XY |
| UPN #169 | F | 57 | MDS-MLD | 47,XX,+11/46,XX |
| UPN #170 | F | 84 | MDS-MLD | 46,XX |
| UPN #171 | F | 84 | MDS-MLD | 46,XX |
| UPN #172 | M | 73 | MDS-MLD | 46,XY |
| UPN #173 | M | 51 | MDS-EB-1 | 47,XY,+8/46,XY |
| UPN #174 | F | 62 | MDS-SLD | 46,XX |
| UPN #175 | F | 78 | MDS with isolated del(5q) | 46,XX,del(5)(q13q31) |
| UPN #176 | M | 79 | MDS-MLD | 46,XY |
| UPN #177 | F | 41 | MDS-MLD | 46,XX |
| UPN #178 | M | 70 | MDS/MPN-U | 46,XY,t(10;22) |
| UPN #179 | M | 74 | MDS-MLD | 46,XY |
| UPN #180 | F | 61 | CMML | 46,XX |
| UPN #181 | M | 67 | MDS-EB-1 | 46,XY |
| UPN #182 | M | 55 | MDS-EB-2 | 46,XY |
| UPN #183 | M | 75 | MDS-MLD | 46,XY |
| UPN #184 | M | 59 | CMML-2 | 45,X,-Y |
| UPN #185 | M | 73 | MDS-RS-SLD | 46,XY |
| UPN #186 | F | 76 | MDS-RS-SLD | 46,XX |
| UPN #187 | M | 82 | CMML-2 | 46,XY |
| UPN #188 | M | 58 | MDS-MLD | 46,XY |
| UPN #189 | M | 79 | MDS-SLD | 46,XY |
| UPN #190 | M | 75 | MDS-MLD | 46,XY |
| UPN #191 | M | 85 | MDS-MLD | 46,XY |
| UPN #192 | M | 51 | MDS-SLD | 46,XY |
| UPN #193 | M | 71 | MDS-MLD | 46,XY |
| UPN #194 | M | 83 | MDS-EB-1 | n.a. |
| UPN #195 | F | 77 | MDS-MLD | 46,XX |
| UPN #196 | M | 67 | MDS-EB-2 | 46,XY |
| UPN #197 | M | 73 | CMML | 45,X,-Y/46,XY |
| UPN #198 | M | 55 | CMML | 46,XY |
| UPN #199 | M | 80 | MDS-MLD | 46,XY |
| UPN #200 | M | 69 | MDS/MPN-U | 46,XY |
| UPN #201 | M | 42 | MDS-MLD | 46,XY |
| UPN #202 | F | 88 | MDS-MLD | 46,XX |
| UPN #203 | M | 74 | CMML-0 | 46,XY |
| UPN #204 | F | 70 | MDS-MLD | 46,XX |
| UPN #205 | M | 75 | MDS-EB-1 | 46,XY |
| UPN #206 | M | 85 | MDS-MLD | 46,XY |
| UPN #207 | M | 62 | MDS-EB-2 | 46,XY |
| UPN #208 | M | 34 | MDS-SLD | 46,XY,del(20)(q)/46,XY |
| UPN #209 | M | 76 | MDS-SLD | 47,XY,+8/46,XY |
| UPN #210 | M | 77 | MDS-MLD | 47,XY,+8/46,XY |
| UPN #211 | F | 82 | MDS-EB-1 | 46,XX |
| UPN #212 | M | 83 | MDS-SLD | 47,XY,+8 |
| UPN #213 | M | 75 | MDS-EB-2 | 47,XY,+8/48,XY,+8,+11/46,XY |
| UPN #214 | F | 72 | MDS-RS-SLD | 47,XX,+8/46,XX |
| UPN #215 | F | 80 | MDS-EB-2 | 47,XX,+8/46,XX |
| UPN #216 | F | 61 | MDS-MLD | 47,XX,+8,del(20)(q11q13) |
| UPN #217 | F | 83 | MDS-U | 47,XX,+8/46,XX |
| UPN #218 | F | 75 | MDS-EB-1 | 47,XX,del(20)(q11q13),+21 /48,idem+8/46,XX |
| UPN #219 | M | 67 | MDS-SLD | 46,XY,del(5)(q31q35)/47,idem,+8/46,XY |
| UPN #220 | M | 91 | MDS-MLD | 45,X,-Y/46,XY |
| UPN #221 | F | 70 | MDS-MLD | 46,XX,del(5)(q13q31)/47,XX,idem,+8/46,XX |
| UPN #222 | F | 81 | MDS-SLD | 46,XX |
| UPN #223 | M | 84 | MDS-EB-2 | 46,XY |
| UPN #224 | M | 68 | MDS-EB-2 | 47,XX,+9/47,idem,del(3)(q12),der(5)del(5)add(5)(q31)/46,XY |
| UPN #225 | F | 64 | MDS-MLD | 46,XY |
| UPN #226 | M | 71 | MDS-SLD | 45,X,-Y/46,XY |
| UPN #227 | F | 65 | MDS with isolated del(5q) | 46,XX,del(5)(q13q31)/46,XX |
| UPN #228 | M | 81 | MDS-MLD | 46,XY |
| UPN #229 | M | 86 | MDS-MLD | 45,X,-Y/ 42-46,XY,idic(1)(q10),der(20)t(1;20)(q11;p11),del(7)(q22)/46,XY |
| UPN #230 | M | 83 | MDS-EB-1 | 47,XY,+14/46,XY |
| UPN #231 | F | 79 | MDS-SLD | 46,XX |
| UPN #232 | M | 49 | MDS-RS-SLD | 46,XY |
| UPN #233 | F | 55 | MDS-EB-2 | 46,XX,del(5)(q13q31) |
| UPN #234 | M | 82 | MDS-EB-1 | 46,XY |
| UPN #235 | M | 72 | MDS-MLD | 45,X,-Y |
| UPN #236 | M | 82 | MDS-EB-1 | 46,XY |
| UPN #237 | F | 66 | MDS-EB-1 | 46,XX,del(5)(q13q31)/46,XX |
| UPN #238 | M | 89 | MDS-EB-1 | 46,XY |
| UPN #239 | M | 73 | MDS-EB-2 | 46,XY |
| UPN #240 | M | 89 | MDS-MLD | 46,XY |
| UPN #241 | M | 81 | MDS-SLD | 47,XY,+15/46,XY |
| UPN #242 | M | 53 | MDS-EB-1 | 44-46,XY,del(5)(q13q35)x2,-7, +8, +mar/46,XY |
| UPN #243 | M | 67 | MDS-RS-SLD | 46,XY,+8/46XY |
| UPN #244 | M | 62 | MDS-EB-2 | 46,XY,t(3;6)(p21;p21)/46,XY |
| UPN #245 | F | 79 | MDS-SLD | 46,XX |
| UPN #246 | F | 87 | MDS-EB-2 | 45-47,XX,+2,-5,del(5)(q31q35),+14,-18,del(20(q11q13),-21,+1-2mar |
| UPN #247 | M | 70 | MDS-SLD | 46,XY,del(20)(q11q13)/45, X,-Y/46XY |
| UPN #248 | F | 82 | MDS-SLD | 46,XX |
| UPN #249 | M | 78 | MDS-MLD | 45,X,-Y |
| UPN #250 | F | 81 | MDS-EB-1 | 45,XX,-7/46XX |
| UPN #251 | F | 85 | MDS-RS-SLD | 46,XY |
| UPN #252 | F | 64 | MDS-EB-2 | 46-,49, XX, del (5)(q31q33), +6, add(6)(p21)X2,+8, +11, -18,+mar/46,XX |
| UPN #253 | M | 66 | MDS-MLD | 45,X,-Y/46,XY |
| UPN #254 | M | 58 | MDS-U | 45,XY,-7/46,XY |
| UPN #255 | M | 94 | MDS-EB-2 | 46,XY |
| UPN #256 | F | 85 | MDS-SLD | 47,XX,+8/46,XX |
| UPN #257 | M | 55 | MDS-U | 46,XY |
| UPN #258 | M | 66 | MDS-EB-2 | 41-48,XY,-3,del(4)(q21q26),del(5)(q13q33),add(6)(q26),-17,+1-2mar/46,XY |
| UPN #259 | F | 66 | MDS-MLD | 46,XX |
| UPN #260 | M | 72 | MDS-EB-2 | 46,XY |
| UPN #261 | M | 81 | MDS-RS-SLD | 45,X,-Y |
| UPN #262 | M | 69 | MDS-MLD | 46,XY,del(20)(q11q13)/46,XY |
| UPN #263 | F | 47 | MDS-SLD | 46,XX |
| UPN #264 | M | 85 | MDS-EB-1 | 47,XY,+8/46,XY |
| UPN #265 | F | 72 | MDS-SLD | 46,XX |
| UPN #266 | F | 75 | MDS-EB-1 | 43,X,der(X)t(X;?)(p?;?),-3,del(5)(q13q34),del(6)(q?),-7,del(7)(q21q34),-11,-12,-12,-17,-17,-18,-19,-20,add(21)(p11),-21,+8mar/46,XX |
| UPN #267 | M | 76 | MDS-EB-1 | 46,XY |
| UPN #268 | M | 59 | MDS-RS-SLD | 46,XY,del(20)(q11q13)/46,XY |
| UPN #269 | F | 72 | MDS-SLD | 46,XX |
| UPN #270 | M | 25 | MDS-EB-1 | 46,XY,t(1;3)(p36;q21),der(6)t(1;6)(q21;q26),add(6)(p24),del(9)(q13q34),add(10)(q26),del(11)(q13q23),del(12)(p11p13),del(20)(q11q13) |
| UPN #271 | F | 88 | MDS-EB-2 | 46,XY |
| UPN #272 | F | 85 | MDS-EB-2 | 46,XX |
| UPN #273 | M | 70 | MDS-EB-2 | 46,XY |
| UPN #274 | M | 66 | MDS-EB-2 | 46,XY,-5,+mar/46,idem,der(1)del(1)(p13),-7,der(10)(?),del(13)(q12q31)+mar |
| UPN #275 | M | 48 | MDS-EB-2 | 46,XY |
| UPN #276 | M | 69 | MDS-RS-SLD | 46,XY,del20(q11q13) |
| UPN #277 | F | 55 | MDS with isolated del(5q) | 46,XX,del(5)(q13q31) |
| UPN #278 | M | 75 | MDS-SLD | 46,XY |
| UPN #279 | M | 78 | MDS-RS-SLD | 46,XY |
| UPN #280 | M | 49 | MDS-SLD | 46,XY |
| UPN #281 | M | 90 | MDS-MLD | 46,XY,der(19)add(19)(p13)/46,XY |
| UPN #282 | M | 62 | MDS-SLD | 46,XY,del(11)(q13q24)/46,XY |
| UPN #283 | M | 72 | MDS-EB2 | 46,XY |
| UPN #284 | M | 82 | MDS-RS-SLD | 46,XY |
| UPN #285 | M | 88 | MDS-MLD | 46,XY |
| UPN #286 | M | 34 | MDS-EB-2 | 46,XY |
| UPN #287 | F | 87 | MDS-SLD | 48,XX,+12,+22/46,XX/46,XY |
| UPN #288 | M | 63 | MDS-RS-SLD | 46,XY,del(20)(q11q13) |
| UPN #289 | M | 83 | MDS-EB-1 | 46,XY |
| UPN #290 | M | 84 | MDS-MLD | 46,XY,del(7)(q22q34),inv(9)(p11q13)/46,XY,inv(9)(p11q13) |
| UPN #291 | M | 72 | MDS-EB2 | 47,XY,+8/46,XY |
| UPN #292 | M | 86 | MDS-MLD | 46,XY |
| UPN #293 | M | 75 | MDS-SLD | 46,XY |
| UPN #294 | M | 75 | MDS-RS-MLD | 46,XY |
| UPN #295 | M | 86 | MDS-SLD | 45,X,-Y/46,idem,+8/46,XY |
| UPN #296 | M | 64 | MDS-EB-1 | 45,XY,-5,-7,add(12)(p13),-18,add(19)(q11),+2mar/46,XY |
| UPN #297 | M | 84 | MDS-MLD | 46,XY |
| UPN #298 | F | 82 | MDS-MLD | 46,XX |
| UPN #299 | F | 83 | MDS-EB-1 | 46,XX,del(5)(q13q34)/46,idem,del(9)(q11q34)/46,XX,del(5)(q13q34),del(11)(q13q23)/46,XX |
| UPN #300 | M | 68 | MDS-EB-1 | 46,XY |
| UPN #301 | M | 81 | MDS-MLD | 46,XY |
| UPN #302 | M | 65 | MDS-EB2 | 46,XY,del(5)(q31q34)/46,idem,del(3)(p21)/48,XY,+1,del(4)(q12q21),del(5)(q31q34),der(7)del(7)(p11)add(7)(q34),+8,del(8)(p11),del(12)(p11p13),add(20)(q13) |
| UPN #303 | M | 66 | CMML-2 | 46,XY,idic(21)(p11.2)/47,idic(21)(p11.2)X2/46,XY |
| UPN #304 | F | 64 | MDS-MLD | 46,XX |
| UPN #305 | M | 64 | MDS-EB2 | 46,XY |
| UPN #306 | F | 70 | MDS-EB2 | 46,XX,t(3;3)(q21;q26) |
| UPN #307 | F | 79 | MDS with isolated del(5q) | 46,XX,del(5)(q31q34)/46,XX |
| UPN #308 | M | 85 | MDS-EB2 | 46,XY,del(5)(q31q34),del(7)(q22q34),-17,del(20)(q11q13)/46,XY |
| UPN #309 | M | 73 | MDS-MLD | 46,XY |
| UPN #310 | M | 58 | MDS-MLD | 46,XY,del(13)(q12q14)/46,XY,inv(11)(p15q13)/46,XY |
| UPN #311 | F | 63 | MDS-EB-1 | 46,XX,del(5)(q13q34)/47,XX,+8/46,XX |
| UPN #312 | F | 67 | MDS-MLD | 46,XX |
| UPN #313 | M | 58 | MDS-MLD | 46,XY,del(1)(p13p31)/46,XY |
| UPN #314 | M | 78 | CMML-2 | 46,XY |
| UPN #315 | M | 71 | MDS-EB2 | 47,XY,+8/46,XY |
| UPN #316 | F | 59 | MDS-MLD | 46,XX |
| UPN #317 | M | 82 | CMML-1 | 45,X,-Y |
| UPN #318 | M | 65 | MDS-MLD | 46,XY |
| UPN #319 | M | 78 | MDS-MLD | 46,XY,der(9)del(9)(q11q34) |
| UPN #320 | M | 76 | MDS-EB-1 | 46,XY |
| UPN #321 | M | 84 | MDS-SLD | 46,XY |
| UPN #322 | F | 86 | MDS-EB2 | 46,XX,del(5)(q31q34)/46,XX |
| UPN #323 | M | 77 | MDS-EB2 | 46,XY,der(1)t(1;13)(p36;q13),+8,del(11)(q23),-13/46,XY |
| UPN #324 | F | 65 | MDS-EB-1 | 46,XX,del(20)(q11q13)/46,XX |
| UPN #325 | M | 65 | MDS-RS-MLD | 46,XY,der(20)del(20)(?)/46,XY |
| UPN #326 | F | 81 | MDS-EB2 | 46,XX,del(20)(q11q13)/46,idem,idic(17)(q10)/46,XX |
| UPN #327 | M | 86 | MDS-EB2 | 47,XY,+8/46,XY |
| UPN #328 | M | 83 | MDS-SLD | 46,XY |
| UPN #329 | F | 78 | CMML-1 | 46,XX |
| UPN #330 | F | 59 | MDS-EB-1 | 43-48,XX,-3,der(5)del(5)t(5;?)(q11;?),-7,+8,add(16)(q22),+21,+21,add(21)(p11),+1-2mar/46,XX |
| UPN #331 | F | 77 | MDS-MLD | 46,XX |
| UPN #332 | M | 66 | CMML-1 | 46,XY |
| UPN #333 | M | 69 | MDS-MLD | 46,XY |
| UPN #334 | M | 64 | MDS/MPN-U | 46,XY |
| UPN #335 | F | 66 | MDS/MPN-U | 46,XX |
| UPN #336 | M | 74 | CMML-1 | 46,XY |
| UPN #337 | M | 65 | MDS-EB1 | 46,XY |
| UPN #338 | F | 79 | CMML-2 | 46,XX |
| UPN #339 | F | 80 | CMML-1 | 46,XX |
| UPN #340 | M | 52 | MDS-MLD | 46,XY |
| UPN #341 | F | 83 | MDS-MLD | 46,XX/46,X,idic(X)(q13) |
| UPN #342 | M | 83 | CMML-2 | 46,XY |
| UPN #343 | M | 73 | CMML-1 | 46,XY |
| UPN #344 | M | 88 | CMML-2 | 46,XY |
| UPN #345 | M | 85 | MDS-MLD | 46,XY |
| UPN #346 | M | 85 | MDS-MLD | 45,X,-Y /46,XY |
| UPN #347 | M | 48 | MDS/MPN-U | 46,XY |
| UPN #348 | F | 90 | MDS-MLD | 46,XX |
| UPN #349 | M | 70 | MDS-MLD | 46,XY |
| UPN #350 | M | 81 | MDS-EB1 | 46,XY |
| UPN #351 | M | 72 | CMML-1 | 46,XY |
| UPN #352 | M | 79 | MDS-RS-MLD | 45,X-Y/46,idem,+8/46,XY |
| UPN #353 | F | 72 | MDS-MLD | 45,XX,-7/46,XX |
| UPN #354 | M | 76 | MDS-MLD | 45,X,-Y/46,XY |
| UPN #355 | F | 47 | MDS-MLD | 46,XX |
| UPN #356 | F | 70 | MDS-RS-SLD | 45,X,-X/44-46,idem,-X,del(1)(q31),del(3)(p13p21),add(8)(p21),+1-2mar/46,XX |
| UPN #357 | F | 85 | CMML-1 | 46,XX |
| UPN #358 | M | 69 | CMML-2 | 46,XY |
| UPN #359 | F | 67 | MDS-SLD | 46,XX |
| UPN #360 | M | 84 | MDS-MLD | 46,XY |
| UPN #361 | F | 68 | MDS-MLD | 46,XX,del(5)(q31q34)/46,XX |
| UPN #362 | M | 70 | MDS-EB1 | 46,XY,del(12)(p11p13)/47,idem,+8/46,XY |
| UPN #363 | M | 85 | MDS-RS-SLD | 46,XY,del(20)(q11q13)/46,XY |
| UPN #364 | M | 70 | MDS-EB1 | 45-46,XY,del(1)(q22q42),add(4)(q?),-5,del(7)(p13),del(13)(q12q14),del(20)(q11q13),+mar/46,XY |
| UPN #365 | M | 63 | MDS-EB1 | 46,XY,der(16)t(16;21)(q12;q11)/46,XY |
| UPN #366 | M | 83 | MDS-EB1 | 47,XY,+8 |
| UPN #367 | F | 62 | MDS-SLD | 46,XX,inv(9)(p13;q11) |
| UPN #368 | M | 81 | MDS-MLD | 46,XY,del(7)(q22q34)/46,XY |
| UPN #369 | M | 24 | MDS-EB1 | 46,XY,del(11)(q21q24) |
| UPN #370 | M | 85 | MDS-MLD | 46,XY |
| UPN #371 | M | 75 | MDS/MPN-U | 46,XY |
| UPN #372 | M | 79 | CMML-0 | 46,XY |
| UPN #373 | F | 63 | MDS-MLD | 46,XX |
| UPN #374 | F | 82 | MDS-MLD | 46,XX |
| UPN #375 | F | 87 | MDS-EB1 | 46,XX |
| UPN #376 | M | 76 | MDS/MPN-U | 46,XY |
| UPN #377 | M | 71 | MDS-RS-SLD | 46,XY,?der(21)(?)/46,XY |
| UPN #378 | M | 79 | MDS-MLD | 45,X,-Y/46,XY |
| UPN #379 | F | 71 | MDS-RS-MLD | 46,XX |
| UPN #380 | M | 60 | MDS/MPN-U | 46,XY |
| UPN #381 | M | 52 | MDS-EB1 | 46,XY |
| UPN #382 | M | 68 | MDS-EB1 | 46,XY |
| UPN #383 | F | 76 | MDS-SLD | 46,XX |
| UPN #384 | M | 79 | MDS/MPN-U | 46,XY |
| UPN #385 | M | 65 | CMML-1 | 46,XY |
| UPN #386 | M | 72 | MDS/MPN-U | 46,XY |
| UPN #387 | M | 72 | MDS/MPN-U | 46,XY |

UPN, unique patient number; F, female; M, male; MDS-EB-1, Myelodysplastic Syndrome with Excess of blasts type 1; MDS-MLD, MDS with multilineage dysplasia; MDS-EB-2, MDS-EB type 2; MDS-SLD, MDS with single lineage dysplasia; MDS-RS-SLD, MDS-SLD with ring sideroblasts; MDS-U, MDS unclassifiable, MDS/MPN-U, MDS/Myeloproliferative neoplasms unclassifiable; CMML, Chronic Myelomonocytic Leukemia; CMML-0, CMML type 0; CMML-1, CMML type 1; CMML-2, CMML type 2, n.a. not available.

**Supplementary Table 3. List of consulted population, disease-specific and sequence databases. Last access March 9^th^ 2020.**

**Supplementary Table 4. Bioinformatic analyses of sequences surrounding the *TERT_P_* new/rare variants using JASPAR Database.** Significantly increased and decreased probability of binding to specific transcription factors are highlighted in red and green, respectively. New binding sites created by the c.1-110_1-101dup are reported in blue.

**Supplementary Figure 1. Family tree of patient UPN#42 (Supplementary Table 2) carrying *TERT_P_* c.1-78T>C variant.** Squares indicate males, circles females, barred squares and circles deceased family members, black square and arrow patient UPN#42 (Supplementary Table 2). Numbers in brackets report each subject’s age at the time of data collection. The genotype (red +/-) indicates *TERT_P_* c.1-78T>C variant in proband and 6 family members who were healthy carriers (age range 18-73 years, mean age 47.5, median 52). The genotype (black +/+) shows no variant was detected in 2 family members.


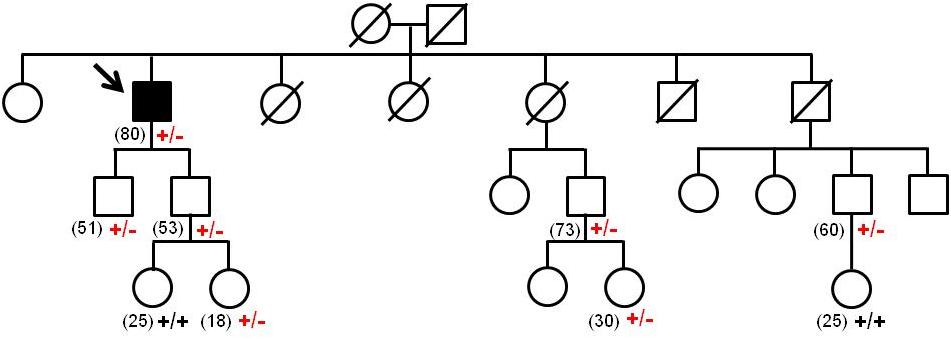


**Supplementary Figure 2. Intra-individual TL in patient UPN#203 (Supplementary Table 2) as analysed by Q-FISH on unstimulated BM cells and PHA-stimulated PB metaphases.** TL is expressed as T/C%. Data are reported as Mean±SD in 4 independent experiments. p<0.05 (Mann-Whitney U test).

**
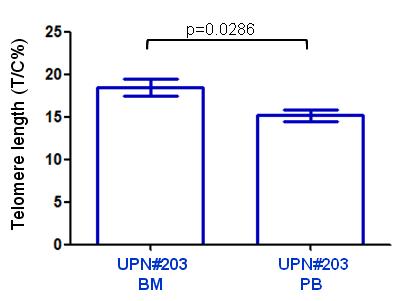
**
